# Model-Based 3D Gaze Estimation Using a TOF Camera

**DOI:** 10.3390/s24041070

**Published:** 2024-02-06

**Authors:** Kuanxin Shen, Yingshun Li, Zhannan Guo, Jintao Gao, Yingjian Wu

**Affiliations:** 1 School of Chemical Process Automation, Shenyang University of Technology, Liaoyang 111003, China; skx13188644041@smail.sut.edu.cn (K.S.); jintao.sut.edu.cn@smail.sut.edu.cn (J.G.); yingjianwu@smail.sut.edu.cn (Y.W.); 2 School of Control Science and Engineering, Dalian University of Technology, Dalian 116024, China; guozhannan@mail.dlut.edu.cn

**Keywords:** model-based gaze estimation, TOF camera, YOLOv8 neural network, eye tracking on driver, low-resolution infrared image

## Abstract

Among the numerous gaze-estimation methods currently available, appearance-based methods predominantly use RGB images as input and employ convolutional neural networks (CNNs) to detect facial images to regressively obtain gaze angles or gaze points. Model-based methods require high-resolution images to obtain a clear eyeball geometric model. These methods face significant challenges in outdoor environments and practical application scenarios. This paper proposes a model-based gaze-estimation algorithm using a low-resolution 3D TOF camera. This study uses infrared images instead of RGB images as input to overcome the impact of varying illumination intensity in the environment on gaze estimation. We utilized a trained YOLOv8 neural network model to detect eye landmarks in captured facial images. Combined with the depth map from a time-of-flight (TOF) camera, we calculated the 3D coordinates of the canthus points of a single eye of the subject. Based on this, we fitted a 3D geometric model of the eyeball to determine the subject’s gaze angle. Experimental validation showed that our method achieved a root mean square error of 6.03° and 4.83° in the horizontal and vertical directions, respectively, for the detection of the subject’s gaze angle. We also tested the proposed method in a real car driving environment, achieving stable driver gaze detection at various locations inside the car, such as the dashboard, driver mirror, and the in-vehicle screen.

## 1. Introduction

Gaze-estimation technology has been receiving increasing attention due to its significant application value. This technology has been applied in numerous fields such as computer communication and text input [1], human–computer interaction [2], virtual reality (VR) [3], psychological research [4], advertising and entertainment gaming [5], animation creation [6], and driver distraction monitoring [7]. Eyeball gazing is a form of non-verbal communication. By observing the gaze points of people in images or scenes, key information hidden in the images can be revealed. Before the application of convolutional neural networks (CNNs) in gaze estimation, the primary research methods for gaze estimation were traditional machine learning and methods based on eyeball geometric models. Mora et al. [8] used gaze appearance images as ground truth images, from which they built appearance models and used regression and KNN machine-learning methods to determine the gaze angle of the subjects. Wood [9] used 3D head scans and realistic rendering methods to create a synthetic eye image dataset and trained a gaze-estimation model on this dataset using machine-learning methods. Chen et al. [10] and Li et al. [11] created realistic 3D eyeball models through facial landmarks detection, allowing for 3D gaze estimation with just a single calibration from the subject. Their 3D eye model does not employ the corneal reflection points of external light sources mentioned by Guestrin et al. [12], a method which is capable of estimating the point of gaze only when the head is held completely still. Furthermore, 3D eye-modeling techniques that utilize corneal reflection points are often considered to be intrusive and unfriendly to the subject, as they constitute an invasive approach to gaze estimation.

Following the rapid advancements in the research and application of convolutional neural networks (CNNs) in the fields of computer vision and deep learning, numerous scientists have begun to incorporate CNNs into the domain of gaze estimation. Zhang et al. [13] proposed the first appearance-based deep gaze-estimation method, and MPIIGaze [14] has become a commonly used dataset for gaze estimation among researchers. Subsequently, Zhang et al. introduced a gaze-estimation method based on the full-face images of subjects [15]. Unlike Ref. [13], which only used the eye region images of the subjects as the model input, Ref. [15] used the entire face image as input, achieving more accurate gaze estimation on the MPIIGaze dataset than [13]. Park et al. [16] proposed a novel 3D gaze image representation method. They used a deep convolutional neural network architecture to handle the projections of the eyeball and iris in 3D space onto a two-dimensional map, and regressed on the map to obtain the gaze angle. Bâce et al. [17] combined appearance-based gaze estimation with optical flow in the eye region to jointly analyze single-channel eye movement dynamics.

In the current landscape of appearance-based gaze estimation, the most advanced model on the MPIIGaze dataset is FAZE [18], while the L2CS [19] model achieved the second-highest global performance ranking on both the Gaze360 and MPIIGaze datasets. These models perform exceptionally well in scenarios using visible light cameras. However, in situations such as nighttime driving tasks, models trained on RGB images prove to be ineffective. As Murthy et al. [20] noted, existing appearance-based gaze-estimation models lack generalization in driving tasks and have not been evaluated for deployment in real-world applications. Vasli et al. [21] proposed a geometry-based gaze-estimation method for drivers, utilizing the physical constraints of the automotive cockpit environment. However, their model, like that of Vora et al. [22], focuses on determining the gaze region of the driver rather than providing a specific and precise gaze angle. Shah et al. [23] believe that using visual area analysis to determine whether a driver is distracted is a fundamental concept. Their work achieved high precision in detecting the gaze angle of drivers in both vertical and horizontal directions. However, it is regrettable that they still only used visible light cameras. Vicente et al. [24] conducted commendable early research. They used a near-infrared camera and a visible light camera to track the driver’s line of sight and evaluated the effects during both day and night. They particularly considered obstructions from sunglasses and extreme head pose. Their work was meticulous and reliable.

**Figure 1:**
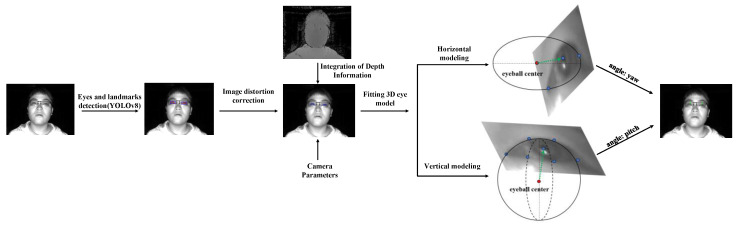
The overall process of the proposed model-based 3D gaze estimation using a TOF camera method. The green arrow represents the subject’s gaze direction.

In contrast to the aforementioned studies, our proposed method for 3D gaze estimation, suitable for automobile drivers, exclusively utilizes infrared images. This approach is instrumental in overcoming the challenges posed by variations in lighting intensity in practical application scenarios, particularly enhancing its utility during nighttime. Considering economic factors in real-world applications, unlike [24], we do not employ multiple cameras, rather, we utilize a single time-of-flight (TOF) camera equipped with an infrared sensor. This study aims to explore a method for implementing 3D gaze estimation on low-resolution TOF cameras (see Figure 1) and to evaluate its accuracy and feasibility in real automotive driving situations. We trained a model using YOLOv8 to detect eye regions and landmarks to obtain the positions of eye landmarks including the pupils and left and right eye corners of the subjects. Then, we performed image distortion correction, followed by the calculation of the 3D coordinates of these eye landmarks using the depth image captured by the TOF camera. We then fit a 3D eyeball model in both horizontal and vertical directions, and on this basis, calculated the final gaze angle. Our primary contributions include:

- We propose a novel method for fitting a 3D eye model. This approach calculates the 3D center of the eyeball using just two eye corners of a single eye. By employing fewer constraint conditions, it reduces computational burden;
- We propose an infrared image dataset for gaze estimation, named “Infrared Gaze Dataset (IRGD)”, created using a TOF camera with a low resolution of 300,000 (640 × 480) pixels and a precision of 1% within a working range of 1 m. This dataset features a larger gaze angle range than MPIIGaze, with participants freely moving their heads during the recording process. Our work aims to achieve gaze estimation under natural conditions;
- Using mean absolute error (MAE) and root mean square error (RMSE) as performance evaluation metrics, we assessed the gaze-estimation method presented in this paper on the benchmark dataset IRGD. We verified that the maximum absolute deviation in gaze angle detection of the proposed model is less than 9 degrees. Furthermore, the model’s reliability in detecting the driver’s line of sight angles was validated in real driving tasks with Toyota commercial vehicles.

The remainder of this paper is organized as follows: Section 2 reviews existing gaze-estimation techniques. Section 3 provides a detailed introduction to our proposed IRGD dataset, comparing it with other publicly available gaze-estimation datasets. Section 4 elaborates on our gaze-estimation method using a TOF camera, including the computation for image distortion correction and detailed modeling of the eyeball model when subjects gaze in horizontal and vertical directions. Section 5 presents the accuracy and evaluation results of the gaze-estimation method proposed in this paper. We also ran five existing state-of-the-art gaze-estimation models on the benchmark dataset IRGD and compared their results with our work. Finally, we collected images of driver gaze during real driving tasks in a Toyota commercial SUV, and evaluated the detection results of the proposed model on these images. Section 6 concludes our work and provides prospects for further research.

## 2. Related Works

In this chapter, we briefly reviewed existing gaze-estimation methods and works related to the method or content of this paper, especially the facial landmarks detection and eyeball center localization methods.

### 2.1. Appearance-Based Gaze Estimation

Different classifications of existing gaze-estimation methods will yield different results when viewed from different angles. From the principle of gaze-estimation models, existing methods can be divided into model-based methods and regression-based methods [25], and regression-based methods can be further subdivided into feature-based methods and appearance-based methods. Feature-based methods learn the mapping function from eye features to gaze direction, with the offset vector between the inner eye corner and the pupil center being the most commonly used feature mapping. However, researchers have discovered a fact: when the tester looks in different directions, the inner eye corner will move [26]. Therefore, the inner eye corner cannot serve as a stable anchor point. Some researchers use the nostril position as the feature vector for regression, but thus far no researchers have confirmed which set of facial feature points is the most ideal [9]. Appearance-based methods learn the mapping relationship from the appearance of the eye to the gaze point or gaze angle, avoiding feature tracking. Park et al. [27] believe that there is a strong relationship between what the user is looking at and the appearance of the user’s eyes. At present, the latest appearance-based methods are almost all implemented based on CNNs. Another study by Park et al. used a CNN architecture to train a stacked hourglass network on the synthetic image dataset UnityEyes to estimate eye area feature points, and on this basis, the gaze direction was obtained through regression [28]. However, as they stated, the most effective appearance-based methods are black-box solutions and understanding why and when they work may be a challenge. Furthermore, although the human eye indeed has three degrees of freedom of motion, due to the physiological relationship between the human eye and the head, people always turn their heads before moving their eyes when they gaze in a certain direction. Therefore, appearance-based gaze estimation is easily affected by head pose.

### 2.2. Model-Based Gaze Estimation

Compared with appearance-based methods, model-based methods can obtain more accurate gaze estimates, but they rely on high-resolution images to fit and track local features used to estimate geometric parameters [29]. A common practice is to use a high-resolution infrared camera to capture the corneal reflection points (Purkinje images) of the subject because the pupil of the eyeball can reflect infrared light well and thus the pupil position can be easily identified on infrared images [30]. By fitting the eyeball with the pupil and corneal reflection points, the direction of the subject’s gaze can be detected with high accuracy on high-resolution infrared images.

Scientists introduced the concepts of visual axis and optical axis to analyze the gaze direction of the human eye. The angle between the visual axis and the optical axis is called the kappa angle, which is approximately 5°. Traditional model-based gaze-estimation methods need to use this physical relationship between the optical axis and the visual axis to solve for the visual axis. Chen et al. [10] proposed a model-based 3D gaze-estimation eyeball modeling method. They used a generic 3D face model composed of six rigid facial points to obtain the 3D coordinates of the subject’s facial landmarks and then calculated the 3D coordinates of the canthus midpoint, eyeball center point, pupil point, and corneal center point of the subject’s single eye in order to calculate the optical axis, and then calculate the 3D visual axis and gaze point position. LI et al. [11] used a research method similar to [10]. Although this traditional model-based gaze-estimation method avoids the intrusion on the subjects as in [12], their solution process is very complex and computationally intensive. Taking the work of [10] as an example, in the calibration process of the subject, for N calibration points, there are 7N equations, with 6N+5 unknowns. Compared with these previous works, we only use the two canthus points of the subject’s single eye to approximate the position of the center of the 3D eyeball. This simple solution method is feasible in our research.

In subsequent model-based gaze-estimation methods, the gaze vector is defined as a line passing through the center of the eyeball to the iris [31]. This definition ignores the kappa angle between the optical axis and the visual axis, omits the calculation of deriving the visual axis from the optical axis, and is equally effective in detecting the gaze direction of the subject. The advantage of the model-based method is that it can consider changes in head pose more effectively than appearance-based methods [30]. This paper, like [8], uses a camera with depth information for gaze estimation under free head movement. The difference is that the depth camera we use also has infrared images.

### 2.3. Facial Landmarks Detection

In the current research, a commonality between model-based methods and appearance-based methods is the need for facial landmarks detection on the subject before gaze estimation. The purpose of detecting facial landmarks in appearance-based gaze-estimation methods is to calculate the head pose of the subject [30] and to standardize the image in conjunction with the head pose before inputting the subject’s gaze image into the neural network [13,15,32,33], to reduce the negative impact of head pose on gaze estimation. Ref. [15] used Alexnet to perform appearance-based full-face gaze estimation on 37,667 images with facial landmarks, Ref. [30] used the open-source model of OpenFace [34] to detect the facial landmarks of the subjects, and ref. [28] used the Tensorflow framework to train a stacked hourglass network for detecting eye region feature points on the annotated synthetic dataset UnityEyes.

The purpose of facial landmarks detection in model-based gaze-estimation methods is to fit and solve the positions of each eye component. Ref. [24] uses the SDM tracker to detect the positions of seven eye landmarks, including the pupil point, on a single-eye image of the subject and calculates the 3D coordinates of the pupil based on this. In existing research methods for estimating the gaze of car drivers, Ref. [23] uses an improved YOLOv4 face detector to detect the driver’s face. The YOLO series of neural networks are one-stage object-detection models, which are widely used in the field of artificial intelligence due to their fast detection speed, high accuracy, and ease of deployment. Unlike [23], this paper uses the open-source YOLOv8-pose [35] module to train a model for detecting the eye area and landmarks of the subject in infrared images on the IRGD dataset labeled with eye corner points, pupil points, etc.

YOLOv8 is an open-source convolutional neural network encompassing functionalities for object detection, instance segmentation, and landmark detection. By default, it utilizes the SiLU (sigmoid linear unit) activation function. The architecture of YOLOv8 comprises two primary components: the backbone and the head. The backbone, a modified version of CSPDarknet53, primarily serves as a feature extractor, responsible for deriving features from the input images. The head, consisting of multiple convolutional layers followed by a series of fully connected layers, is tasked with predicting the bounding boxes, object detection scores, and class probabilities for detected objects within the image. YOLOv8 employs CIoU (complete intersection over union) and DFL (distance-IoU front-end loss) for bounding box location loss, alongside BCE (binary cross-entropy) for classification loss. The network is standardly configured with 53 layers. In this study, the YOLOv8s neural network was utilized to train a subject’s eye landmark detection model. The images input into the network were of a default size of 640 × 480 pixels. Upon invoking the trained landmark detection model for inference on single-subject gaze images within the test set, the model outputs a tensor of dimensions [2,27], where 2 corresponds to the detected left and right eyes of the subject, while 27 represents the detected attributes of a single eye. These attributes include the bounding box’s position (x, y, w, h), the confidence of the bounding box, the probabilities associated with the categories, and the 2D coordinates and confidences of seven landmark points.

#### Data Augmentation

**Figure 2:**
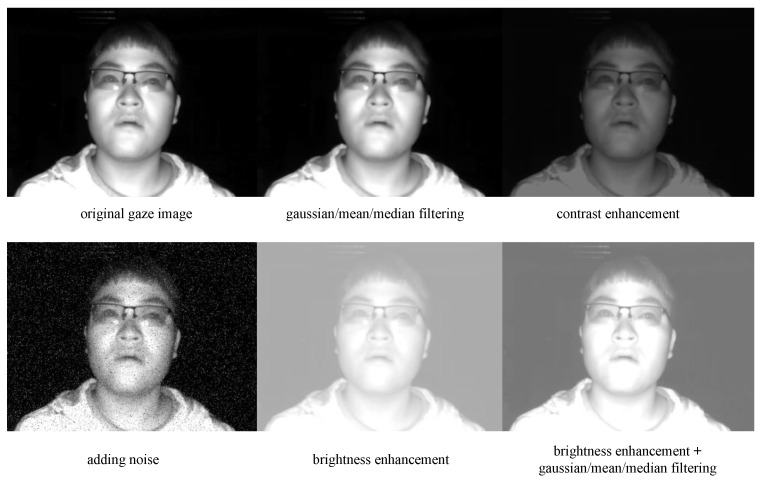
The partial effectiveness of data augmentation.

Data augmentation is an effective method to prevent overfitting in eye landmark detection models. As illustrated in Figure 2, we augment the images from the IRGD dataset using computer vision methods such as Gaussian filtering, mean filtering, median filtering, brightness enhancement, contrast enhancement, adding salt and pepper noise, horizontal flipping, rotation, and scaling. Through this approach, the amount of data used to train the eye landmark detection model in this study was increased by a factor of six from the original 75,000 images in the IRGD dataset, resulting in a total of 525,000 images. Furthermore, this paper divides the dataset into a training set and a testing set in a 4:1 ratio. These data augmentation techniques serve as a regularization mechanism for training neural network models in facial landmark detection. During the training process, data augmentation introduces randomness into the data fed into the network. This randomness is then mitigated in the test set to enhance the network’s generalization ability. Implementing this practice is expected to improve the robustness of the facial landmark detection model in complex scenarios with variable lighting conditions.

**Figure 3:**
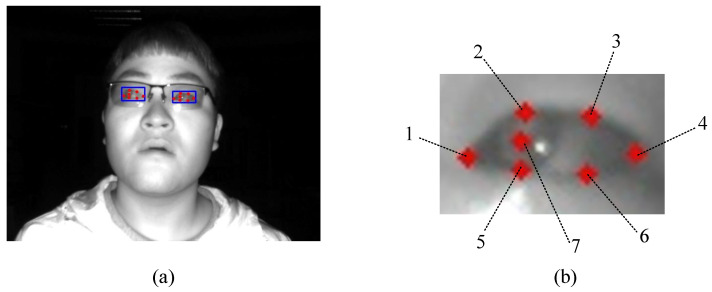
The eye region and landmark detection model trained on the IRGD dataset using YOLOv8 shows the detection effect on the subject’s gaze image (**a**). The landmark detection model outputs 7 target points for a single-eye image of the subject (**b**): 1—Left eye corner point; 2—First upper eyelid point; 3—Second upper eyelid point; 4—Right eye corner point; 5—First lower eyelid point; 6—Second lower eyelid point; 7—Pupil point.

The eye landmarks detection model we trained, based on YOLOv8, outputs the coordinates of seven eye landmarks for a single eye image of the subject, including the left and right eye corners, the pupil, and the upper and lower eyelid points (see Figure 3). To improve work efficiency, we annotate the eye landmarks of participants on the IRGD dataset in a semi-automatic manner to quickly obtain training set data.

### 2.4. 3D Eyeball Center Positioning Method

The center of the eyeball is an eye component hidden outside the image, which cannot be directly observed in the gaze image like the eyelids and pupils. Existing model-based gaze-estimation literature varies in their computation and fitting methods for the 3D center of the eyeball. Refs. [10,11] detect the head pose angle and sequentially calculate the 3D pupil point coordinates and 3D corneal center point coordinates in the facial landmarks, then obtain the 3D coordinates of the eyeball through individual calibration of the subject. Their calculation method is quite complex. Vicente et al. [24] assumed that the eyeball is spherical and is at a rigid point relative to the head model, thereby approximating the 3D coordinate position of the eyeball. However, this is merely an approximate estimation method and unfortunately, they have not proven the effectiveness of their method.

#### 2.4.1. Localization Method Based on Eye Corners and Eyeball Radius

Modern medical data on human eyeball anatomy indicate that the average radius of the human eye is 12 mm. In model-based gaze estimation, a novel method for locating the eyeball center involves obtaining the 3D coordinates of the left and right eye corners to compute the 3D midpoint of the eye corners. The eyeball center is then defined as the position 12 mm away from the eye corner midpoint in the *Z*-axis direction of the camera coordinate system, thereby transforming the model-based method into a calibration-free gaze-estimation method. Ref. [30] used the open-source OpenFace model to detect the head pose of the subject, combined with the 3D coordinates of the left and right eye corners and the constant value of the 12 mm eyeball radius to calculate the 3D coordinates of the subject’s eyeball center. The subject’s gaze angle is then computed based on this.

**Figure 4:**
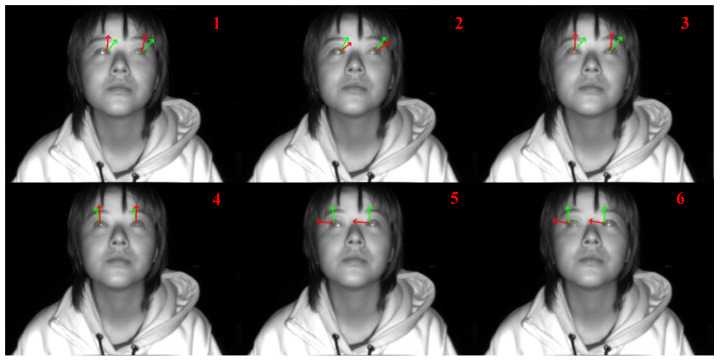
The subject maintained a head pose angle of 0° in both the horizontal and vertical directions and performed a series of coherent lizard movements. The green arrow indicates the ground-truth gaze direction, while the red arrow represents the final gaze direction obtained using the eyeball center calculation method proposed in [30]. As the subject’s gaze angle gradually increased, the deviation between the gaze angle calculated by this eyeball center localization method and the ground-truth gaze angle began to increase. Table 1 shows the results of our calculations.

We replicated the eyeball center localization method mentioned in [30]. To eliminate the interference of head pose on the eyeball center localization method mentioned in [30], we calculated the horizontal gaze angle (yaw) on the gaze images of the subject’s lizard movement collected by the TOF camera (see Figure 4). The feasibility of this 3D eyeball center localization method was judged by the absolute deviation size between the calculated gaze angle and the ground-truth angle. We manually annotated the left and right eye corner points and pupil points of these six images and strictly calculated the 3D eyeball center coordinates according to the eyeball center localization method of [30]. We found that within ±30° of small gaze angle calculation, the eyeball center localization method mentioned in [30] can obtain very accurate gaze estimation, with an absolute deviation angle value within 5°. However, this eyeball center localization method fails in gaze estimation at large angles greater than 30°.

#### 2.4.2. 3D Eyeball Fitting Based on Nonlinear Least Square Method

**Figure 5:**
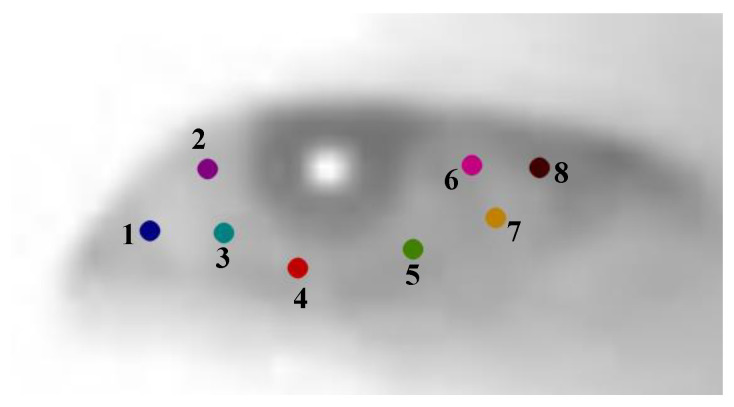
Eight marked points are manually annotated on the image of the subject’s single eye. These points are randomly distributed on the sclera of the eye, not the cornea. We use these eight 3D coordinate points to fit the eyeball model and solve for the 3D coordinates of the eyeball center and the radius of the eyeball.

In the many methods of fitting the 3D eyeballs, we noticed a type of nonlinear least squares method. Like most studies [21,24], we assume that the eyeball is spherical. We replicated the method of fitting the eyeball in [36], solving for the coordinate values of the 3D eyeball center through eight marked points on a single-eye image (see Figure 5), with the number of marked points satisfying the condition of array size ≥ 5 required for the solving process. We collected images at approximate distances of 200 mm, 300 mm, and 400 mm from the TOF camera to the subject’s head, and the radius values of the fitted 3D eyeball models are shown in Table 2.

**Figure 6:**
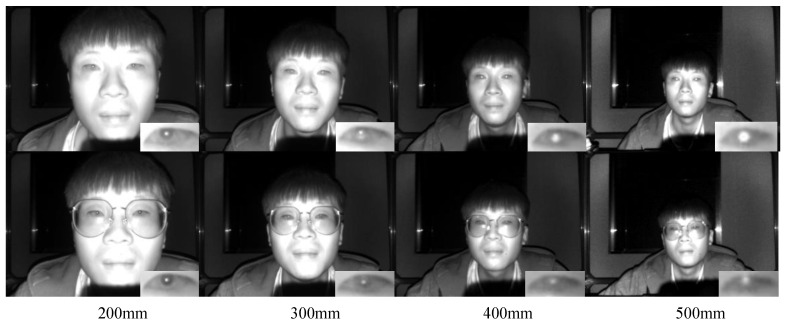
Eye detail images taken by the TOF camera at a distance of 200 mm–500 mm from the subject. The experiment is divided into two scenarios: the subject not wearing myopia glasses (**top**) and wearing glasses (**bottom**). The occlusion of glasses reduces some of the clarity and contrast of the subject’s eyes, but it is much less than the impact of a longer distance. When the distance between the subject and the TOF camera exceeds 300 mm, the only observable details in the eye area are the corners of the eyes and the pupil points.

The nonlinear least squares method has demonstrated highly accurate fitting results in fitting the 3D eyeball model. As shown in Table 2 the fitted eyeball radius is very close to the average value of 12 mm of the human eye. However, it relies on high-resolution eye images and a large number of marked points. The more marked points involved in fitting the eyeball and the more dispersed the distribution of marked points on the sclera, the more accurate the solved eyeball model. However, obtaining as many marked points as possible on low-resolution images is a challenge. We collected detailed images of the eyes of subjects wearing glasses and not wearing glasses at different distances between 200 mm and 500 mm from a 300,000-pixel TOF camera (see Figure 6). When the distance between the subject’s eyes and the TOF camera exceeds 300 mm, the contrast between the sclera and cornea severely decreases, and the only easily observable details in the subject’s eye area are the corners of the eyes and the pupils. Therefore, the eyeball center locating method based on nonlinear least squares does not apply to the low-resolution infrared images used in this study. We propose a method to fit the 3D eyeball center using only the left and right corner points of a single eye (see Section 4.3). The subject only needs to undergo one calibration to complete the solution calculation.

## 3. Dataset

To achieve stable 3D gaze estimation under various complex backgrounds and lighting conditions, we used a TOF camera equipped with an infrared sensor to record the benchmark dataset IRGD for gaze estimation. The use of an infrared camera can overcome the negative impact of uneven changes in lighting intensity on gaze estimation. The gaze-estimation model based on infrared images can operate in two different scenarios, day and night, which is extremely beneficial for the gaze estimation of car drivers and has been reflected in the work of [24].

### 3.1. Existing Dataset

Currently, many public datasets for gaze estimation have been generated in previous studies. Due to the different purposes and focuses of these studies, the characteristics of their public datasets also vary. Siegfried et al. [37] researched gaze estimation in conversation and operation scenarios and recorded the Gaze_VFOA dataset, which is a video dataset including three sub-datasets such as ManiGaze [38]. The ManiGaze dataset is especially used for assessing the gaze estimation of subjects during human–computer interaction. Other video datasets include EYEDIAP [39] and EVE [27], of which EVE was recorded on a computer monitor using a network port camera, aiming to estimate the gaze of subjects watching videos. Kellnhofer et al. recorded the large-scale gaze tracking dataset Gaze360 [40] which includes indoor and outdoor environments. They used the AprilTag image that was also used in the ManiGaze dataset during the recording process, which is convenient for marking target locations in 3D space. Similar to Gaze360, the MPIIGaze dataset is also used for gaze estimation in outdoor environments. This dataset is collected from subjects gazing at laptop screens in daily life. Due to the limitations of the recording scenario, the gaze angle range of the MPIIGaze dataset is relatively small. Due to the privacy protection set by the authors, researchers can only access the images of the eye area in the MPIIGaze dataset. If researchers want to explore the role of information from other parts of the face in gaze estimation, they can access the MPIIFaceGaze dataset, which comes from the same source as MPIIGaze. The latter is based on the former and provides complete facial area images and facial feature point annotations. For research on gaze estimation in extreme head poses, the ETH-XGaze dataset is often used. This dataset provides high-resolution images under extreme head pose and gaze directions with over a million samples [32]. The ETH-XGaze dataset contains images with a resolution of 24 MP (6000 × 4000 pixels). Another dataset with high-resolution images is Columbia [41], with an image resolution of 18 MP (5184 × 3456). However, the Columbia dataset only has 5880 images, and compared to the MPIIGaze dataset, Columbia has a smaller gaze angle range. Krafka et al. proposed a large-scale dataset, GazeCapture [42], for eye-tracking on mobile phones and tablets. The gaze-estimation model, iTracker, they developed based on the GazeCapture dataset was used in a commercial mobile phone software. Because this work is based on gaze estimation on small planes such as mobile phones or tablets, GazeCapture, like MPIIGaze, has the disadvantage of a smaller gaze angle range.

Synthetic datasets are another type of dataset commonly used in gaze-estimation work. Unlike real human eye gaze images, synthetic datasets are based on the scanning and reconstruction of real human eyes using 3D rendering models. However, this does not mean that synthetic datasets are not practical. In fact, synthetic datasets avoid the angle errors in the recording process of real eye datasets. For example, in our research experience, due to physiological factors, the subjects’ eyes inevitably become tired during the long data-recording process, leading to errors in gaze angle. The recording process of real human eye datasets requires the subjective cooperation of the subjects. Sugano et al. [33] proposed a synthetic dataset, UT Multi-view, which contains multi-view gaze images reconstructed from real human eyes. Other synthetic datasets include SynthesEyes and UnityEyes. SynthesEyes contains 11,382 synthesized close-up eye images and 10 dynamic eye area models; these synthesized images have achieved photo-realistic quality [43]. The UnityEyes dataset is generated by rendering 3D eye area models, capturing a large number of eye appearance variations, and can estimate challenging eye gaze images [44]. Wood has created open-source software to generate the UnityEyes dataset, and researchers can use this software to generate synthetic eye images with custom resolutions.

Most existing popular gaze datasets are RGB images with high resolution. However, image blur is a factor that leads to inaccuracies in the analysis of human social interactions involving gaze [45]. Therefore, it is particularly necessary to explore gaze estimation on low-resolution images. The low-resolution IR image dataset proposed in this paper addresses the challenge of RGB images in existing public datasets being easily affected by lighting conditions. A summary and comparison of the popular open-source datasets and the IRGD dataset proposed in this study can be seen in Table 3.

### 3.2. Data Collection Procedure

The TOF camera can simultaneously capture infrared images and depth images. The infrared images provide 2D information about the human eye during the gaze process, while the depth images provide 3D information about the 2D eye landmarks in the camera coordinate system. TOF cameras have been widely used in the automotive intelligent cockpit industry for driver behavior detection such as smoking and phone calling. To save economic costs for car manufacturers, we perform model-based 3D gaze estimation based on the automotive-grade TOF camera with a resolution of 300,000 pixels and an accuracy of 1% (1 m), which is widely used in cars.

**Figure 7:**
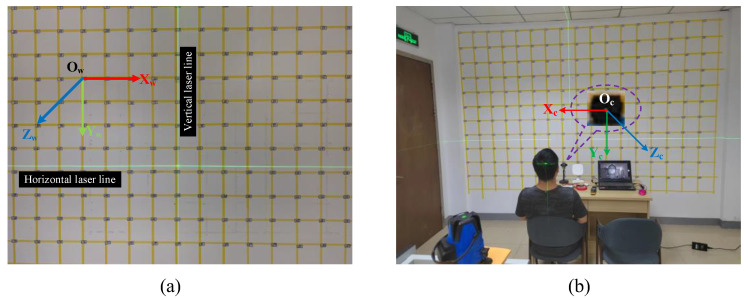
Creating a standard plane with multiple gaze points using a level’s laser line (**a**) and fixing a TOF camera on the plane (**b**).

Before officially recording the IRGD dataset, we created and calibrated a standard plane for gaze estimation using a level, based on a standard wall (see Figure 7a). This standard plane consists of a grid of squares with a side length of 200 mm, arranged in 17 columns and 11 rows. We ordered the grid, and the intersections of the grid on the standard plane are the gaze points, which are located in the coordinate system of the standard plane. To increase the diversity of lighting conditions during data recording, we chose the location of the standard plane at the indoor doorway to approach the lighting changes in the external environment and organized participants to record data at three different times: morning, noon, and evening. The TOF camera is fixed on the standard plane and does not change its position during data recording. The size of the standard plane is 3.4 m × 2.2 m, which is sufficient to meet the recording of gaze data at extreme angles. We created a standard plane coordinate system $X_{w}-Y_{w}-Z_{w}$ and camera coordinate system $X_{c}-Y_{c}-Z_{c}$ (see Figure 7b). Since the fixed position of the camera on the standard plane is known, the 3D coordinate values of each gaze point in the camera coordinate system can be obtained through the transformation relationship between the two coordinate systems. The specific transformation calculation is as follows:

(1) $$P_{x}=-\left(s\times \left(num-\left(col\times \lfloor \frac{num}{col}\rfloor \right)-1\right)-camera_{x}\right)$$

(2) $$\;\;\;\;\;\;\;\;\;\;\;\;\;\;\;\;\;\;\;\;\;\;\;P_{y}=s\times \lfloor \frac{num}{col}\rfloor -camera_{y}$$

(3) $$P_{z}=-camera_{z}$$

where $\left(P_{x},\;P_{y},P_{z}\right)$ represents the 3D coordinates of the gaze point on the standard plane in the coordinate system of the TOF camera. $\left(camera_{x},camera_{y},camera_{z}\right)$ denotes the fixed position of the TOF camera in the coordinate system of the standard plane. s represents the size of the regular quadrilateral grid on the standard plane, which is set to 200 mm in this study. num represents the gaze point number, and col represents the number of regular quadrilateral grids per row on the standard plane.

**Figure 8:**
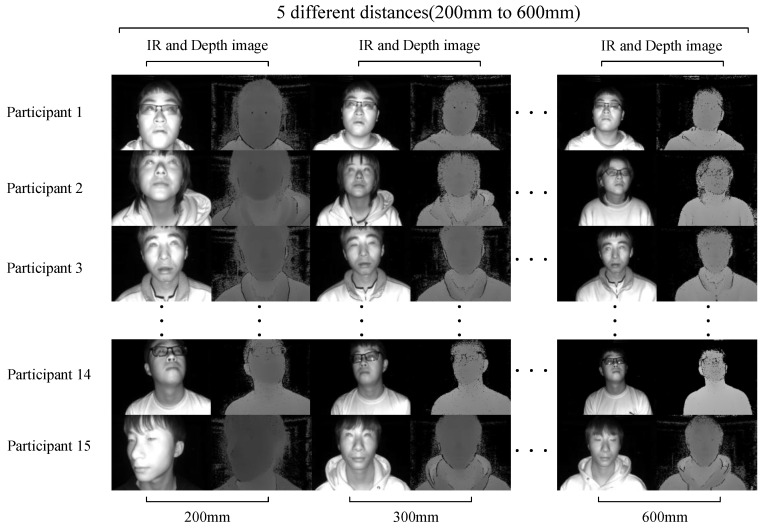
Sample pictures of the IRGD dataset proposed in this paper. We recorded gaze data at five different distances from the participant to the TOF camera, ranging from 200 mm to 600 mm. The TOF camera simultaneously collected IR images and depth images of the participant gazing at the gaze points on the standard plane. All participants performed natural eye movements and coherent head movements.

To establish a tacit understanding with the participants during the data-recording process, we developed a camera acquisition software and ran it on a laptop. Unlike Columbia and UT Multi-view, we did not use a head fixation device, allowing participants in the data recording to perform natural eye movements as described in the EVE dataset, without any constraints. Participants freely gaze at the gaze points on the standard plane and inform the camera acquisition software of the current gaze point number via voice commands. The camera acquisition software captures the participant’s gaze image 0.5 s later. Figure 8 shows some examples from the IRGD dataset we recorded.

### 3.3. Dataset Characteristics

The process of recording the dataset was quite laborious [28], and the creation of the IRGD dataset proposed in this paper took a considerable amount of time. We ultimately organized 15 participants to join this work, including 7 individuals wearing nearsighted glasses, 9 males, and 6 females, all of whom were Asian. Each participant recorded data at five different distances from the TOF camera: 200 mm, 300 mm, 400 mm, 500 mm, and 600 mm (see Figure 8). The TOF camera had a sampling frame rate of 30 fps and a sampling interval of 4, capturing 1000 IR images and 1000 depth images for each participant at each distance, resulting in a total of 75K IR images and their corresponding 75K depth images in the IRGD dataset. These ample data laid a solid foundation for training the eye landmarks detection model using YOLOv8. To provide a comprehensive description of the characteristics of the IRGD dataset, we discuss it in detail from the following aspects.

#### 3.3.1. Image Resolution

The IRGD dataset proposed in this paper includes both IR and depth images. These two types of images were captured by an automotive-grade TOF camera with 300,000 pixels, both of which have a low resolution of 640 × 480.

#### 3.3.2. Gaze Direction

The IRGD dataset is composed of gaze images from 15 participants, each of whom sequentially gazed at 35 target gaze points on a standard plane. These gaze points form a gaze area of 1.4 m × 0.8 m on the standard plane, with the participants’ gaze angles in the horizontal and vertical directions within this gaze area being ±50°.

#### 3.3.3. Light Condition

In this study, a lux meter was used to record the ambient illumination levels during data acquisition. To enhance the adaptability of the gaze-estimation model to variations in illumination intensity, the benchmark dataset IRGD was recorded under a wide range of lighting conditions, from complete darkness (0 lux) to illumination levels nearing outdoor sunlight exposure (170,000 lux). The automotive-grade TOF camera utilized in this work demonstrated stable performance across this spectrum of illumination intensities.

#### 3.3.4. Head Pose

**Figure 9:**
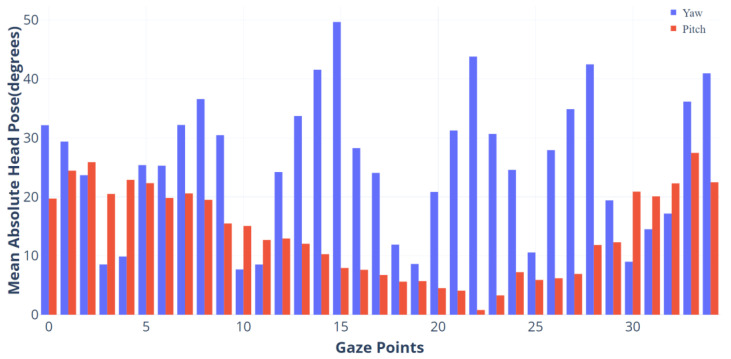
The absolute values of the average head pose angles of the participants at 35 gaze points in the IRGD dataset. The maximum absolute angle of the participants’ head pose in the horizontal direction (yaw) is approximately 50°, while in the vertical direction (pitch), it is approximately 30°.

Existing work on head pose handling can be divided into two categories: one is to normalize the images using the detected head pose angles before training the network to reduce the impact of head pose on gaze estimation [15], and the other is to use head pose to compensate for the estimated gaze angle [24]. The head pose handling approach Mora et al. mentioned is more ingenious than that of [15]; they use an RGBD camera and rigid ICP algorithm to track the subject’s head pose frame by frame to generate the subject’s frontal gaze image. This method has the advantage of decoupling the gaze angle from the head pose, significantly reducing the complexity of gaze-estimation problems caused by the head pose [8]. However, for cases where the subject’s eyes are self-occluded at larger gaze angles, it is not possible to restore the subject’s frontal gaze image from the gaze image with the head pose. The gaze-estimation method proposed in this paper calculates the subject’s eyes in 3D space, so it is not disturbed by head pose like traditional 2D-image-based gaze-estimation methods. Our work does not directly regress gaze angles from gaze images, so there is no need to normalize the images like [33]. Since head pose detection is not the goal of this study, we only use existing head pose detection methods to statistically analyze the head pose angles of the IRGD dataset. We use the 6-point facial method [46] and the 12-point method [47] to detect the head poses of participants in the manually annotated IRGD dataset, but the detected results have significant deviations in both direction and angle. Subsequently, we used the state-of-the-art head pose estimation model 6DRepNet [48] on the BIWI dataset to detect and calculate the absolute values of the average head pose angles of participants gazing at 35 gaze points in the IRGD dataset (see Figure 9).

## 4. Method

In this chapter, we will focus on the distortion correction calculations on the IR gaze images taken by the TOF camera and the methods of fitting the 3D eyeball center in the horizontal and vertical directions. We will discuss the drawbacks of using the TOF camera directly for gaze estimation and the methods to address this issue.

### 4.1. Image Distortion Correction

Distortion refers to the degree of distortion of the image formed by an optical system relative to the object itself. It is an inherent characteristic of optical lenses, directly caused by the inconsistent magnification between the edge and the center of the lens. Radial distortion is caused by the shape of the lens, while tangential distortion is caused by the tilt of the lens and the imaging plane during the camera assembly process [49]. Thanks to the improvement of camera assembly and production technology, the distortion values of current industrial cameras and network cameras are relatively small, with the former generally having a distortion value of only 1–2 pixels, and the latter only a few pixels. The average distortion value of the TOF camera used in this study is 7 pixels, with different distortion sizes at different positions on the image, and the smallest distortion value in the central area. For gaze-estimation methods on 2D images, image distortion may not affect the accuracy of gaze estimation like other factors. However, our work requires extracting the depth value at the pixel coordinates of the target point on the depth image, and the depth values of different target points may not be the same. Before correcting the distortion of the eye landmarks on the subject’s gaze image, we use OpenCV’s camera calibration tool [50] to obtain the intrinsic matrix and distortion coefficients of the TOF camera:

(4) $$Intrinsic\;Matrix=\left[\begin{array}{ccc} f_{x} & 0 & c_{x}\\ 0 & f_{y} & c_{y}\\ 0 & 0 & 1 \end{array}\right]$$

(5) $$Distortion\;Coefficient=\left(k_{1},k_{2},p_{1},p_{2},k_{3}\right)$$

where $\left(k_{1},k_{2},k_{3}\right)$ represents the radial distortion coefficient, and $\left(p_{1},p_{2}\right)$ denotes the tangential distortion coefficient.

In Section 2.3, we presented the output results of the eye landmarks detection model trained using YOLOv8. For the 7 landmarks obtained on the single-eye image of the subject shown in Figure 3b, the calculation methods for radial and tangential distortion correction are as follows:

(6) $$r_{i}^{2}=u_{i}^{2}+v_{i}^{2}$$

(7) $$\overset{´}{u_{i}}=u_{i}*\left(1+k_{1}r_{i}^{2}+k_{2}r_{i}^{4}+k_{3}r_{i}^{6}\right)+2p_{1}u_{i}v_{i}+p_{2}\left(r_{i}^{2}+2u_{i}^{2}\right)+u_{i}$$

(8) $$\overset{´}{v_{i}}=v_{i}*\left(1+k_{1}r_{i}^{2}+k_{2}r_{i}^{4}+k_{3}r_{i}^{6}\right)+2p_{1}\left(r_{i}^{2}+2v_{i}^{2}\right)+2p_{2}u_{i}v_{i}+v_{i}$$

where $\left(u_{i},v_{i}\right)$ represents the 2D pixel coordinates of the i-th target point among the 7 landmarks detected on the original gaze image, and $\left(\;\overset{´}{u_{i}},\overset{´}{v_{i}}\right)$ denotes the pixel coordinates of the i-th landmarks point after distortion correction. We only correct the distortion of the eye landmarks in the subject’s gaze image, rather than every pixel of the image. This approach is beneficial for improving the computational efficiency of gaze estimation.

### 4.2. Integration of Depth Information

After eye landmarks detection and distortion correction on the IR gaze images captured by the TOF camera, we utilize the depth information from the TOF camera to convert the coordinates of the landmarks in the 2D image coordinate system into 3D coordinates in the camera coordinate system. The detailed calculation method is as follows:

(9) $$X_{i}=\frac{Z_{i}*\left(\overset{´}{u_{i}}-\frac{w}{2}\right)}{f_{x}}$$

(10) $$Y_{i}=\frac{Z_{i}*\left(\overset{´}{v_{i}}-\frac{h}{2}\right)}{f_{y}}$$

(11) $$Z_{i}=\frac{g_{i}}{255}*\left(f-n\right)+n$$

where $\left(X_{i},Y_{i},Z_{i}\right)$ is the 3D coordinate value corresponding to the 2D pixel coordinate $\left(\overset{´}{u_{i}},\overset{´}{v_{i}}\right)$ of the i-th eye landmark, $f_{x}$ and $f_{y}$ are the intrinsic parameters of the TOF camera. w and h are the width and height of the image, which are 640 and 480, respectively, in this study. $g_{i}$ is the grayscale value at point $\left(\overset{´}{u_{i}},\overset{´}{v_{i}}\right)$ on the depth image, f is the maximum distance value of the TOF camera’s range measurement, and n is the minimum distance value of the TOF camera’s range.

### 4.3. Fitting of Eyeball

**Figure 10:**
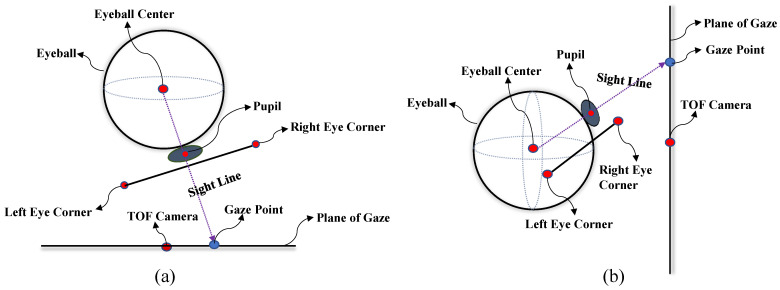
Independent modeling and solution of eyeball center coordinates in horizontal (**a**) and vertical (**b**) gaze directions of subjects.

In this study, we fit the center of the eyeball using the left and right canthus points on the image of a single eye of the subject. We draw inspiration from Abdelrahman et al. [19] who used two fully connected layers to independently regress each gaze angle (yaw and pitch) to improve the prediction accuracy of gaze estimation. We model the eyeball in the horizontal (yaw) and vertical (pitch) directions when the subject is gazing (see Figure 10). We use the coordinates of the subject’s eyeball center fitted in the horizontal and vertical directions to calculate the gaze angles in the two directions, respectively. This study assumes that the left and right canthus points of the subject are on the spherical surface with the eyeball center as the center and the distance to the surface of the eyeball is dif. For the center of the eyeball in the horizontal direction, its Y-coordinate value is set as the average of the left and right canthus points. Assuming that the radius of the eyeball model in the horizontal direction $R_{1}$ and dif are known, the detailed calculation of the center of the eyeball is as follows:

(12) $$y_{eye}=\frac{Y_{1}+Y_{2}}{2}$$

(13) $${\left(x_{eye}-X_{1}\right)}^{2}+{\left(y_{eye}-Y_{1}\right)}^{2}+{\left(z_{eye}-Z_{1}\right)}^{2}={\left(R_{1}+dif\right)}^{2}$$

(14) $${\left(x_{eye}-X_{4}\right)}^{2}+{\left(y_{eye}-Y_{4}\right)}^{2}+{\left(z_{eye}-Z_{4}\right)}^{2}={\left(R_{1}+dif\right)}^{2}$$

where $\left(X_{1},Y_{1},Z_{1}\right)$ and $\left(X_{4},Y_{4},Z_{4}\right)$ are the 3D coordinates of the left and right canthus points of the subject after distortion correction, respectively. $\left(x_{eye},y_{eye},z_{eye}\right)$ is the calculated center coordinate of the subject’s eyeball in the horizontal gaze direction in the TOF camera coordinate system, and $z_{eye}$ is taken as the maximum value solution in the calculation.

**Figure 11:**
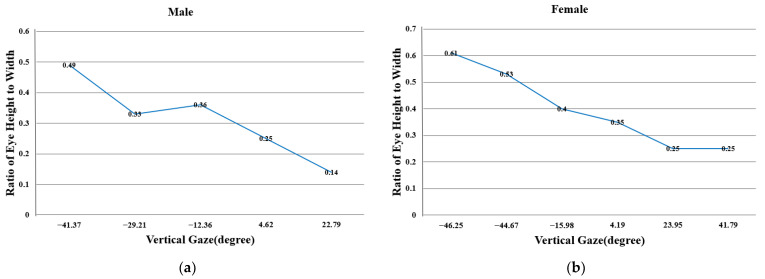
Variation trends of the aspect ratio of eye appearance with vertical gaze angle in male (**a**) and female (**b**) participants. In male participants, the aspect ratio of eye appearance is less than 0.3 when the eyeball is looking down, while in female participants, the aspect ratio of eye appearance is less than 0.4 when the eyeball is looking down.

For the vertical direction of the eyeball model, the X-coordinate value of the eyeball center is set as the average of the left and right eye corner points. The following method is used to solve for the position of the eyeball center:

(15) $$\overset{´}{x_{eye}}=\frac{X_{1}+X_{4}}{2}$$

(16) $${\left(\overset{´}{x_{eye}}-X_{1}\right)}^{2}+{\left(\overset{´}{y_{eye}-}Y_{1}\right)}^{2}+{\left(\overset{´}{z_{eye}}-Z_{1}\right)}^{2}={\left(R_{2}+dif\right)}^{2}$$

(17) $${\left(\overset{´}{x_{eye}-}X_{4}\right)}^{2}+{\left(\overset{´}{y_{eye}}-Y_{4}\right)}^{2}+{\left(\overset{´}{z_{eye}-}Z_{4}\right)}^{2}={\left(R_{2}+dif\right)}^{2}$$

where $R_{2}$ represents the radius value of the participant’s eyeball when gazing in the vertical direction. Assuming $R_{2}$ is known, the vertical coordinate $\left(\overset{´}{x_{eye}},\overset{´}{y_{eye}},\overset{´}{z_{eye}}\right)$ of the eyeball center can be calculated. In fact, in the specific calculation process, the value of $\overset{´}{y_{eye}}$ depends on the participant’s vertical eyeball pitch state. If the coordinate axis direction of the TOF camera coordinate system $X_{c}-Y_{c}-Z_{c}$ in Figure 7b is used to define the positive and negative values of the gaze angles in horizontal and vertical directions, then $\;\overset{´}{y_{eye}}$ takes the minimum value solution when the participant is looking down (pitch in the positive direction), and $\overset{´}{y_{eye}}$ takes the maximum value solution when the participant is looking up. This study uses the aspect ratio of the participant’s eye appearance to determine the pitch state of the eyeball. We randomly selected participants for experiments to explore the trend of changes between the vertical gaze angle pitch of the eyeball and the aspect ratio p of the participant’s eye appearance. Figure 11 presents our experimental results.

From the research results of Baltrušaitis [51], we find that for gaze-estimation tasks, the accuracy of gaze angle detection in the vertical direction of the participants is lower than that in the horizontal direction, which is a typical phenomenon in gaze estimation. The reason for this phenomenon is that there are fewer pixels on the iris used for *Y*-axis estimation, and the pupil being covered by the upper and lower eyelids is also a significant factor. Therefore, it is highly meaningful to study the gaze angles of the participants in the horizontal and vertical directions separately. The work of predecessors has validated the value of our research.

### 4.4. Compute the Aspect Ratio of Eye Appearance

The purpose of calculating and statistically analyzing the aspect ratio of the eye appearance of the participant’s eyes before gaze estimation is to determine the pitch state of the participant’s eyeball in the vertical direction, to obtain the correct solution for the participant’s eyeball center in the vertical direction. This work is primarily accomplished in the calibration phase of Section 4.6. Section 2.3 presents the results of the participant’s eye landmark detection. This paper uses the 2 upper eyelid points and 2 lower eyelid points output from the participant’s single-eye gaze image to calculate the aspect ratio of the participant’s eye appearance. It is worth noting that when manually annotating the training set images for landmark detection, the first upper eyelid point is annotated at 1/3 of the participant’s upper eyelid contour, and the second upper eyelid point is annotated at 2/3 of the upper eyelid contour. Similarly, the first lower eyelid point is annotated at 1/3 of the lower eyelid contour, and the second lower eyelid point is annotated at 2/3 of the lower eyelid contour (see Figure 3b). The eye landmark detection model trained under these annotation position constraints can output 4 eyelid point coordinates that also satisfy the position relationship. This constraint calculates the aspect ratio of the participant’s eye appearance more accurately. The calculation of the aspect ratio of the participant’s eye appearance is as follows:

(18) $$\left\{\begin{array}{c} u_{a}=\frac{\overset{´}{u_{2}}+\overset{´}{u_{3}}}{2},v_{a}=\frac{\overset{´}{v_{2}}+\overset{´}{v_{3}}}{2},Z_{a}=\frac{g_{a}}{255}*\left(f-n\right)+n\\ u_{b}=\frac{\overset{´}{u_{5}}+\overset{´}{u_{6}}}{2},v_{b}=\frac{\overset{´}{v_{5}}+\overset{´}{v_{6}}}{2},\;Z_{b}=\frac{g_{b}}{255}*\left(f-n\right)+n\\ X_{a}=\frac{Z_{a}*\left(u_{a}-\frac{w}{2}\right)}{f_{x}},Y_{a}=\frac{Z_{a}*\left(v_{a}-\frac{h}{2}\right)}{f_{y}}\\ X_{b}=\frac{Z_{b}*\left(u_{b}-\frac{w}{2}\right)}{f_{x}},Y_{b}=\frac{Z_{b}*\left(v_{b}-\frac{h}{2}\right)}{f_{y}}\\ p=\frac{\sqrt{{\left(X_{a}-X_{b}\right)}^{2}+{\left(Y_{a}-Y_{b}\right)}^{2}+{\left(Z_{a}-Z_{b}\right)}^{2}}}{\sqrt{{\left(X_{1}-X_{4}\right)}^{2}+{\left(Y_{1}-Y_{4}\right)}^{2}+{\left(Z_{1}-Z_{4}\right)}^{2}}} \end{array}\right.$$

where $\left(\overset{´}{u_{2},}\overset{´}{v_{2}}\right)$ represents the pixel coordinates of the first upper eyelid point on the distortion-corrected gaze image of the participant, and $\left(\overset{´}{u_{3}},\overset{´}{v_{3}}\right)$ represents the pixel coordinates of the second upper eyelid point after distortion correction. $\left(u_{a},v_{a}\right)$ is the pixel coordinates of the midpoint of the two upper eyelid points. Similarly, $\left(\overset{´}{u_{5},}\overset{´}{v_{5}}\right)$ and $\left(\overset{´}{u_{6}},\overset{´}{v_{6}}\right)$ are the pixel coordinates of the first lower eyelid point and the second lower eyelid point after distortion correction, respectively, and $\left(u_{b},v_{b}\right)$ is their midpoint. $g_{a}$ and $g_{b}$ are the grayscale values of $\left(u_{a},v_{a}\right)$ and $\left(u_{b},v_{b}\right)$ on the depth image of the TOF camera, respectively. $\left(X_{a},Y_{a},Z_{a}\right)$ and $\left(X_{b},Y_{b},Z_{b}\right)$ are the 3D coordinates in the TOF camera coordinate system corresponding to $\left(u_{a},v_{a}\right)$ and $\left(u_{b},v_{b}\right)$, respectively. p is the calculated aspect ratio of the participant’s eye appearance.

### 4.5. Drawbacks of TOF Cameras in Operation

**Figure 12:**
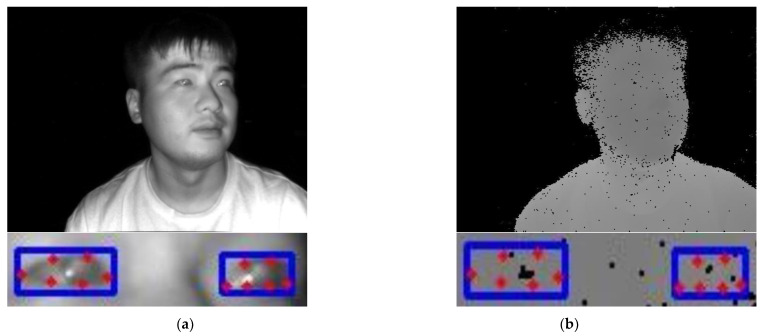
The drawback of the inability to extract pupil point depth values from the depth image of the TOF camera. For gaze images at certain special angles, the pupil point can be observed on its IR image (**a**), but due to the absorption of infrared light by the pupil, a ‘black hole’ appears at the position of the pupil point on the corresponding depth image (**b**) of the IR image.

The infrared light emitted by the TOF camera has strong penetration and can directly illuminate the pupil area. In addition, the pupil reflects infrared light more than the cornea and lens; hence, the pupil presents higher contrast and brightness in the infrared image, making it easy to detect on the infrared image. The TOF camera relies on the infrared pulse light reflected by the object under test to measure the distance from the object to the camera, but at certain special gaze angles, the subject’s pupil will absorb the infrared pulse light from the TOF camera. This results in the pupil being easy to detect on the 2D infrared image but unable to extract depth information from on the depth image (see Figure 12).

This study does not use the method of obtaining distance information of landmarks on the subject’s face using a 3D face model [10,13,15,32], but calculates the depth value under the TOF camera coordinate system for each eye landmark using the grayscale value on the depth image. We found that the depth values of the left and right eye corners of the subject can always be stably obtained. However, in certain special head poses or gaze angles, due to the pupil absorbing the infrared light from the TOF camera, a ‘black hole’ appears at the corresponding position on its depth image. This results in a grayscale value of 0 for the pupil point on the depth image, thereby making it impossible to solve for the 3D coordinate value of the pupil point.

In response to the operational drawback of the TOF camera being unable to extract the depth information of the pupil point in some subjects’ gaze images, we use the constraint relationship that the distance between the subject’s pupil point and the center of the eyeball is a certain value, combined with the 2D pixel coordinates of the pupil point detected on the IR gaze image, to solve for the 3D coordinates of the pupil. Since we model the subject’s horizontal and vertical gaze directions separately, the distance between the pupil point and the center of the eyeball in these two directions is two different values for the subject. Assuming that the distance in 3D space between the pupil point and the center of the eyeball in the horizontal gaze direction of the subject is $d_{1}$, then:

(19) $$\{\begin{array}{c} \begin{array}{c} X_{7}=\frac{Z_{7}*\left(\overset{´}{u_{7}}-\frac{w}{2}\right)}{f_{x}}\\ Y_{7}=\frac{Z_{7}*\left(\overset{´}{v_{7}}-\frac{h}{2}\right)}{f_{y}}\\ {\left(x_{eye}-X_{7}\right)}^{2}+{\left(y_{eye}-Y_{7}\right)}^{2}+{\left(z_{eye}-Z_{7}\right)}^{2}=d_{1}^{2} \end{array} \end{array}$$

where $\left(\overset{´}{u_{7}},\overset{´}{v_{7}}\right)$ represents the pixel coordinate values of the pupil outputted from the eye landmark detection model after distortion correction on the subject’s IR gaze image. $\left(X_{7},Y_{7},Z_{7}\right)$ signifies the calculated 3D coordinate values of the pupil point in the TOF camera coordinate system when the subject gazes in the horizontal direction. The value of $Z_{7}$ is obtained by taking the minimum solution.

Assuming that the distance between the pupil point and the center of the eyeball in the vertical gaze direction of the subject is $d_{2}$, we then have:

(20) $$\{\begin{array}{c} \begin{array}{c} \overset{´}{X_{7}}=\frac{\overset{´}{Z_{7}}*\left(\overset{´}{u_{7}}-\frac{w}{2}\right)}{f_{x}}\\ \overset{´}{Y_{7}}=\frac{\overset{´}{Z_{7}}*\left(\overset{´}{v_{7}}-\frac{h}{2}\right)}{f_{y}}\\ {\left(\overset{´}{x_{eye}}-\overset{´}{X_{7}}\right)}^{2}+{\left(\overset{´}{y_{eye}}-\overset{´}{Y_{7}}\right)}^{2}+{\left(\overset{´}{z_{eye}}-\overset{´}{Z_{7}}\right)}^{2}=d_{2}^{2} \end{array} \end{array}$$

where $\left(\overset{´}{X_{7}},\overset{´}{Y_{7}},\overset{´}{Z_{7}}\right)$ represents the calculated 3D coordinate values of the pupil point when the subject gazes in the vertical direction, and $\overset{´}{Z_{7}}$ is obtained by taking the minimum solution.

After addressing the operational drawbacks of the TOF camera and obtaining the 3D coordinate values of the pupil points under the camera coordinate system, the coordinates of the eyeball center from Section 4.3 can be used to determine the gaze angles yaw and pitch of the subject in the horizontal and vertical directions, respectively:

(21) $$yaw=\frac{180}{\pi }*\tan^{-1}\begin{array}{c} \left(\frac{X_{7}-x_{eye}}{z_{eye}-Z_{7}}\right) \end{array}$$

(22) $$pitch=\frac{180}{\pi }*\tan^{-1}\begin{array}{c} \left(\frac{\overset{´}{Y_{7}}-\overset{´}{y_{eye}}}{\overset{´}{z_{eye}}-\overset{´}{Z_{7}}}\right) \end{array}$$

### 4.6. Person-Dependent Calibration

The average value of the human eyeball radius is 12 mm, and the distance from the center of the eyeball to the pupil is 13.1 mm [11]. Through our measurements, the average value of dif among different subjects is 5 mm. Using these constants to assign values to the above variables and calculate the final gaze angles in the horizontal and vertical directions, we found that precise calculation results can be obtained at the gaze points of individual subjects, but these constants do not apply to most subjects, and the deviation of the calculated gaze angles far exceeds 10°. The calculation of the subject’s eyeball center and pupil in this paper is based on the known conditions of $R_{1}$, $R_{2}$, $d_{1}$, $d_{2}$, and dif. These subject-specific parameters need to be obtained through calibration. The research results of Zhang et al. [13] show that among the various challenges faced by unconstrained gaze, the performance gap in gaze estimation caused by individual differences is 40%, which is higher than the 25% of gaze range and 35% of lighting conditions. Therefore, in model-based gaze estimation, individual calibration is also an important task, which can significantly improve the accuracy of gaze estimation.

**Figure 13:**
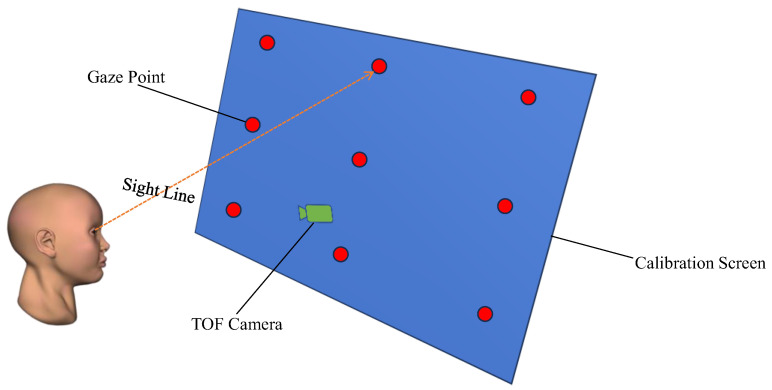
Schematic diagram of the calibration process for individual-specific eyeball parameters of the subject.

Chen et al. [10] proposed a 9-point method for calibrating individual-specific eyeball parameters of subjects, while Shah et al. [23] suggested that users could be instructed to look at specific points on the screen to adjust the intrinsic parameters of the eye model to avoid time-consuming calibration procedures. In the experimental results of [25], the more calibration samples there are, the smaller the average deviation of the gaze angles calculated by the algorithm, and as the number of samples increases, the calibrated eyeball parameters approach the ground-truth parameters. Our work combines their calibration approaches:

1. Assuming there is a calibration screen in the subject’s gaze space and as many gaze points as possible are spread across the calibration screen (see Figure 13). The number of gaze points is denoted as M, and the position relationship of each gaze point with the TOF camera is recorded. The ground-truth gaze angle of the eyeball when the subject gazes at the gaze point in the r-th row and c-th column on the calibration screen in the horizontal direction is denoted as $\alpha_{rc}$, and the ground-truth gaze angle of the eyeball when gazing at the gaze point in the r-th row and c-th column in the vertical direction is denoted as $\beta_{rc}$;
2. Constraining the values of $R_{1}$, $R_{2}$, $d_{1}$, and $d_{2}$ within a certain range and traversing these parameters with a step size of 0.1 within this range, we calculate the absolute deviation between the gaze angle values obtained by each set of parameters at each gaze point and the ground-truth angle values, using the aforementioned gaze-estimation calculation method;
3. Computing the average absolute gaze angle deviation corresponding to each set of parameters at M gaze points in N calibrations. The set of $R_{1}$, $R_{2}$, $d_{1}$, and $d_{2}$ parameters that yield the smallest average absolute deviation is outputted and denoted as the ground-truth parameters of the subject’s eyeball.

In the specific experiment, since the influence of the dif value on the gaze angle is consistent with $R_{1}$ and $R_{2}$, it can be considered that the calibration effect of dif is included in the calibration effect of $R_{1}$ and $R_{2}$. Therefore, we set dif as a fixed value of 5 mm and only calibrate $R_{1}$ and $R_{2}$. From the perspective of human eye anatomy, the parameters of the human eye must have reasonable values [25]. After fully considering the prior values of the structural parameters of the human eye in medicine, we set the values of $R_{1}$, $R_{2}$, $d_{1}$, and $d_{2}$ to [5,40].

In the calibration process, the calculation of the absolute deviation between the gaze angle of the subject’s eyeball obtained by the gaze-estimation method in this paper and the ground-truth angle corresponding to the gaze point on the calibration screen is as follows:

(23) $$e=\left|\alpha_{rc}-yaw_{rc}\left(R_{1}d_{1}\right)\right|,R_{1}\in \left[5,\;40\right],d_{1}\in \left[5,\;40\right]$$

(24) $$\overset{´}{e}=\left|\beta_{rc}-pitch_{rc}\left(R_{2}d_{2}\right)\right|,R_{2}\in \left[5,40\right],d_{2}\in \left[5,\;40\right]$$

where $yaw_{rc}\left(R_{1}d_{1}\right)$ represents, within the range of [5,40] when the horizontal radius of the subject’s eyeball is $R_{1}$ and the distance from the center of the eyeball to the pupil is $d_{1}$, the horizontal gaze angle calculated by the gaze-estimation method proposed in this paper when the subject gazes at the gaze point at the r-th row and c-th column on the calibration screen. Similarly, $pitch_{rc}\left(R_{2}d_{2}\right)$ represents, within the range of [5,40] when the vertical radius of the subject’s eyeball is $R_{2}$ and the distance from the center of the eyeball to the pupil is $d_{2}$, the vertical gaze angle calculated by the gaze-estimation method in this paper when the subject gazes at the gaze point at the r-th row and c-th column. e represents the absolute deviation between the horizontal gaze angle calculated under the current set of $\left(R_{1},d_{1}\right)$ parameters and the ground-truth angle, while $\overset{´}{e}$ represents the absolute deviation between the vertical gaze angle of the subject calculated under the current set of $\left(R_{2},d_{2}\right)$ parameters and the ground-truth angle.

The calculation method for the average gaze-estimation angle deviation of M gaze points on the calibration screen in N calibrations is as follows:

(25) $$\bar{e\left(yaw\right)}=\frac{\sum_{i=1}^{N}\sum_{j=1}^{M}e_{ij}}{N*M}$$

(26) $$\bar{e\left(pitch\right)}=\frac{\sum_{i=1}^{N}\sum_{j=1}^{M}\overset{´}{e_{ij}}}{N*M}$$

where $\bar{e\left(yaw\right)}$ represents the average angle deviation in the horizontal direction, $\bar{e\left(pitch\right)}$ represents the average angle deviation in the vertical direction, $e_{ij}$ represents the absolute deviation between the gaze angle value corresponding to the j-th gaze point on the calibration screen and the ground-truth angle during the i-th calibration by the subject in the horizontal direction, and $\overset{´}{e_{ij}}$ represents the absolute deviation between the gaze angle value corresponding to the j-th gaze point on the calibration screen and the ground-truth angle during the i-th calibration by the subject in the vertical direction.

**Figure 14:**
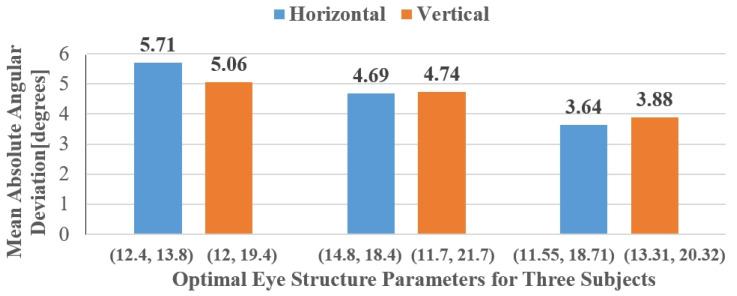
Calibration results of eyeball parameters for three subjects. We obtained the optimal eyeball structure parameters $\left(R_{1},d_{1}\right)$ and $\left(R_{2},d_{2}\right)$ for 3 subjects through 10 calibrations, each involving gazing at 20 gaze points. At the same time, we calculated the mean absolute deviation between the gaze angles in the horizontal direction (blue) and vertical direction (orange) computed from this set of parameters and the ground-truth angles.

In this study, the calibration screen used for calibrating the subject-specific eye structure parameters is not a fixed scene. The subject should choose an appropriate calibration screen based on the actual application of gaze estimation. For example, when implementing gaze estimation for drivers in a car, the car’s central control screen can be used as the calibration screen. When performing eye tracking for gamers on a computer, the computer monitor can be used as the calibration screen. The selection of the calibration screen should be combined with the subject’s work environment, and the subject should perform complete and as many gaze point calibrations as possible. Figure 14 shows the calibration results of the eye structure parameters for three randomly selected subjects.

## 5. Experiment

For the model-based 3D gaze-estimation method presented in this paper, we demonstrate its accuracy and effectiveness in detecting the gaze of subjects through several experiments. Following existing experimental approaches, we evaluate the method using a leave-one-out strategy on one subject from the IRGD dataset, while the data from the remaining 13 subjects are used for training the eye landmark detection model based on YOLOv8. We apply the trained model to detect facial images of the evaluation subject at different distances and calculate the detection deviation of the seven landmarks on the subject’s single-eye image by comparing the Euclidean distance between the detected landmarks’ pixel coordinates and the ground-truth landmarks’ pixel coordinates. The final calculated mean deviation is 7.42 pixels. Since four of the seven landmarks (eyelid points) are only used for determining the subject’s eyeball pitch state, when disregarding the eyelid points detection deviation, the model’s average detection deviation for the two eye corner points and the pupil point used to calculate the gaze angle is 1.86 pixels. Therefore, we believe that a YOLOv8 landmark detection model with a good detection accuracy has been trained on the IRGD dataset.

### 5.1. Pupil Depth Error

**Figure 15:**
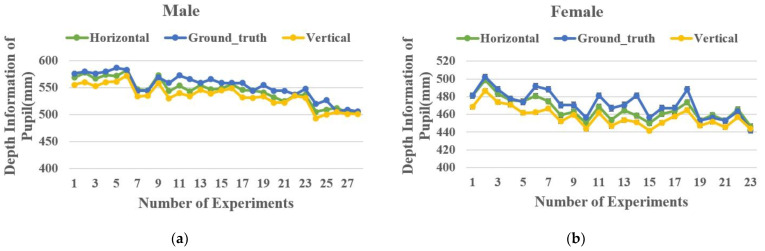
Experiment results on calculating the average pupil depth information and corresponding ground-truth values in horizontal and vertical gaze directions for the male group (**a**) and female group (**b**).

We conducted experiments on the IRGD dataset by dividing it into two different groups, male and female, to test the accuracy of the pupil depth calculation method proposed in this paper. In total, nine male subjects participated in 28 experimental sessions within a range of 500 mm–600 mm from the TOF camera, while six female subjects participated in 23 sessions within a range of 400 mm–500 mm. We intentionally aimed to verify the accuracy of pupil depth calculation at different distances. Figure 15 shows the results of the calculated pupil depth values and the ground-truth pupil depth values obtained during the experiments.

For the male subject group, the mean absolute error between the calculated pupil depth values and the ground-truth pupil depth in the horizontal gaze direction was 9.23 mm, and in the vertical gaze direction, it was 17.81 mm. For the female subject group, the mean absolute error in the horizontal gaze direction was 6.73 mm, and in the vertical gaze direction, it was 14.39 mm. Overall, the pupil depth calculation method proposed in this study has a greater accuracy in the horizontal direction than in the vertical direction.

### 5.2. Deviation of Gaze Angle

**Figure 16:**
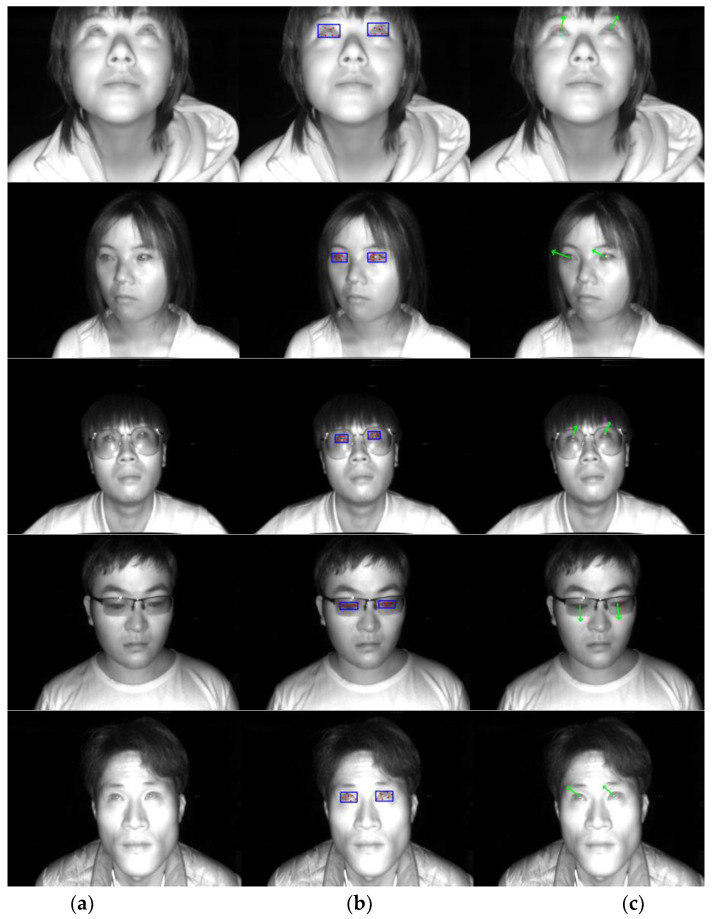
Results of the subject’s gaze detection. Column (**a**) presents the original gaze images of the subject, column (**b**) shows the results of eye landmark detection based on YOLOv8, and column (**c**) visualizes the subject’s gaze direction. The green arrow indicates the gaze direction detected by our model.

**Figure 17:**
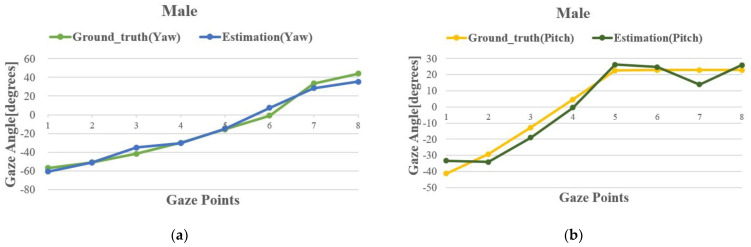

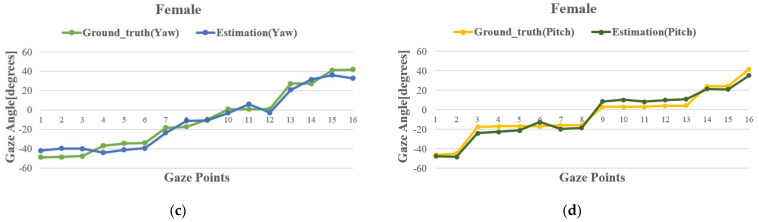
Gaze angle detection results of male and female subject groups using the gaze-estimation method proposed in this study. Specifically, (**a**) represents the horizontal gaze results of the male group, (**b**) shows the vertical gaze results of the male group. (**c**) illustrates the horizontal gaze results of the female group, and (**d**) presents the vertical gaze results of the female subjects.

Figure 16 presents the results of the gaze-estimation method proposed in this paper for the subjects’ eye tracking. The results in Section 2 indicate that when the subjects are closer to the TOF camera, the clarity of the eye region is higher. To evaluate the gaze-estimation model’s detection results at greater distances, we maintained the nine male subjects at a distance of 500 mm–600 mm from the TOF camera and the six female subjects at a distance of 400 mm–500 mm. These test ranges closely approximate the typical distances between drivers and the common camera installation locations in real driving scenarios. Figure 17 shows the comparison between the estimated gaze angles and the ground-truth angles for male subjects targeting 8 uniformly distributed points within the gaze space and female subjects targeting 16 points in the test environment. To evaluate the accuracy of our method in detecting the subjects’ gaze angles, we used the mean absolute error (MAE) as the performance evaluation criterion to calculate the average deviation between the model’s detection results and the ground-truth gaze angles for both male and female subject groups. Ultimately, the average deviation for the male subjects was 4.51° in the horizontal direction and 4.43° in the vertical direction. For the female subjects, the average deviation was 5.71° in the horizontal direction and 5.07° in the vertical direction.

### 5.3. Comparison with Other State-of-the-Art Methods

**Figure 18:**
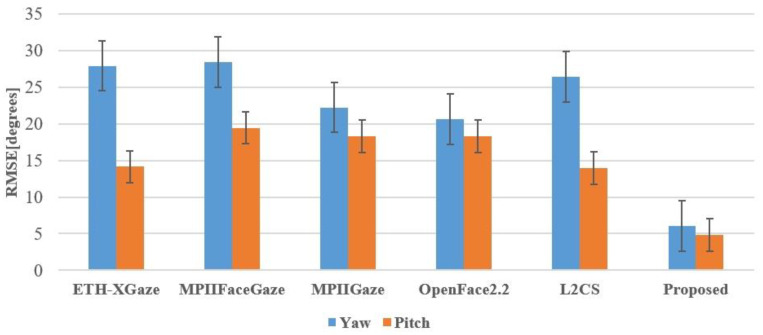
Comparative accuracy results of our proposed gaze-estimation model with other state-of-the-art models in infrared gaze test images.

In this subsection, we combined the test data from both male and female groups and set up a testing environment for state-of-the-art gaze-estimation models such as OpenFace2.2, ETH-XGaze, MPIIGaze (also known as GazeNet), MPIIFaceGaze (also referred to as Spatial Weights CNN), and L2CS on local computer devices. These state-of-the-art models are all trained on RGB images. Kendrick et al. [52] showed that RGB images perform best in facial landmark detection before gaze estimation, and the model converges the fastest. However, when predicting a wide range of landmarks, grayscale images are superior to RGB images. RGB images are sensitive to lighting and partial occlusion, which lack feature points [53]. In our experiments, we found that these state-of-the-art models have issues with infrared image data, as they are unable to detect the facial landmarks of the subjects and thus cannot output gaze angles. OpenFace2.2 performs better in detecting infrared gaze images of subjects than ETH-XGaze, MPIIFaceGaze, and MPIIGaze, as the latter three often fail to output gaze angles. L2CS significantly deviates from the ground-truth size in the angle values detected from some infrared gaze images. We calculated the accuracy of these five state-of-the-art models and the gaze-estimation model proposed in this study based on RMSE (see Figure 18). The gaze-estimation method proposed in this study has a root mean square error of 6.03° in the horizontal direction (yaw) and 4.83° in the vertical direction (pitch). The method for calculating the root mean square error (RMSE) between the gaze angle values detected by the model for the subjects and the ground-truth gaze angle values is as follows:

(27) $$RMSE\left(yaw\right)=\sqrt{\frac{1}{Q}\sum_{i=1}^{Q}{\left(yaw_{i}-yaw_{t,i}\right)}^{2}}$$

(28) $$RMSE\left(pitch\right)=\sqrt{\frac{1}{Q}\sum_{i=1}^{Q}{\left(pitch_{i}-pitch_{t,i}\right)}^{2}}$$

where RMSE(yaw) represents the root mean square error (RMSE) between the model-predicted gaze angle and the ground-truth gaze angle in the horizontal direction, while RMSE(pitch) denotes the RMSE for the vertical direction. Q signifies the number of experimental samples. $yaw_{i}$ refers to the model-predicted gaze angle of the subject in the horizontal direction during the i-th experiment, and $yaw_{t,i}$ is the ground-truth gaze angle of the subject in the horizontal direction. Similarly, $pitch_{i}$ represents the model-predicted gaze angle in the vertical direction for the i-th experiment, and $pitch_{t,i}$ indicates the ground-truth gaze angle of the subject in the vertical direction.

### 5.4. Experiments in Real Driving Scenarios for Automobile

**Figure 19:**
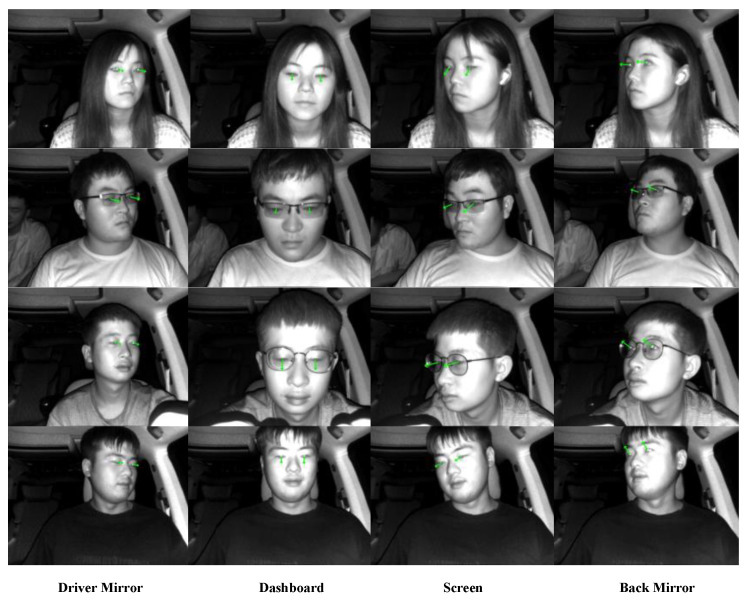
Detection results of driver’s partial gaze points in the interior of a Toyota business SUV. Green arrows indicate the driver’s gaze direction detected by our gaze-estimation model.

**Figure 20:**
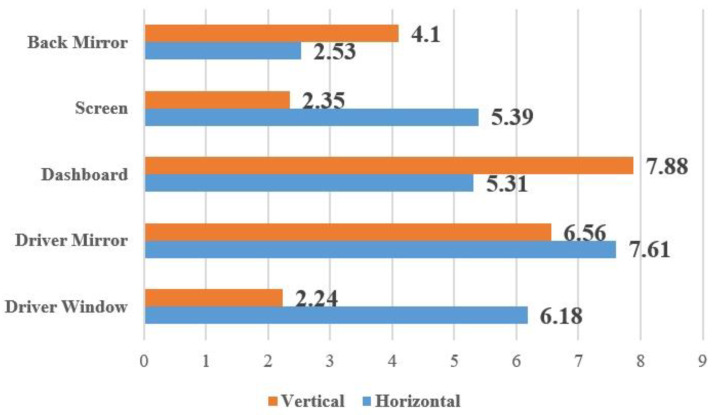
Mean absolute error between the detected driver’s gaze angles and ground-truth angles at various gaze points inside the car.

To validate the performance of our gaze-estimation model in real-world application scenarios, we conducted driver gaze detection in a real driving environment within a full-scale car cabin. We organized 15 subjects to gaze at five target points during car driving, including the dashboard, back mirror, in-vehicle screen, driver mirror, and driver window (see Figure 19). Before recording these gaze data, we first calibrated the five gaze points and the TOF camera fixed points in the Toyota business SUV used, to measure the ground-truth gaze angles of the 15 drivers. The subjects performed free driving tasks in the experiment, with the TOF camera fixed in front of the car steering wheel and at a distance of 450 mm–600 mm from the subjects. Figure 20 shows the mean absolute error between the detection results of our gaze-estimation model for these target points and the ground-truth gaze angles. In normal car driving, the distance from the driver to the dashboard is about 600 mm. Moreover, adjusting the installation position of the TOF camera and maintaining a closer distance between the camera and the driver can appropriately improve the accuracy of driver gaze detection.

### 5.5. Discussion

Calibration of the subject’s eyeball parameters is a very important task for our model-based gaze-estimation method. In the experiment, setting individual subjects’ eyeball radius and the distance from the center of the eyeball to the pupil as medical constants of 12 mm and 13.1 mm, respectively, can also achieve a good gaze angle detection accuracy, but not all subjects’ eyeball parameters are close to these two constants. Therefore, it is imperative to calibrate individual-specific eyeball parameters. Since we approximate the position of the subject’s eyeball center separately in the horizontal and vertical directions, the eyeball structure parameters estimated in these two gaze directions are also different. Summarizing the overall computational process, the implementation steps of the gaze-estimation method proposed in this paper are as follows:

1. Utilizing the YOLOv8s neural network, a model for eye landmark detection is trained. This landmark detection model is then employed to perform inference on the gaze images of subjects, yielding 2D coordinates of seven landmark points on the image of a single eye of the subject, including points at the corners of the eye and the pupil;
2. Distortion correction is applied to the gaze images of the subjects, resulting in the acquisition of the 2D coordinates of the seven eye landmarks after distortion correction;
3. By integrating the depth images captured simultaneously by the TOF camera during the subject’s gaze, the 3D coordinates of the seven landmark points are computed within the coordinate system of the TOF camera;
4. An eyeball model in the horizontal and vertical directions is constructed using the left and right corner points of a single eye of the subject. The aspect ratio of the eye’s appearance during the subject’s gaze is calculated. Based on this aspect ratio value, the solution for the eyeball’s center point in the horizontal and vertical gaze directions of the subject is determined;
5. Based on the distance constraint between the center of the eyeball and the pupil point, combined with the 2D coordinates of the pupil on the gaze image of the subject, the 3D coordinates of the pupil in the horizontal and vertical directions within the TOF camera coordinate system are calculated;
6. Calibration of the subject-specific parameters of the eyeball structure is conducted to ascertain the optimal structural parameters of the eyeball and the range of the subject’s eye appearance aspect ratio variation with the vertical gaze angle. Based on these optimal parameters, the gaze angles of the subject in the horizontal and vertical directions, specifically yaw and pitch, are calculated.

**Figure 21:**
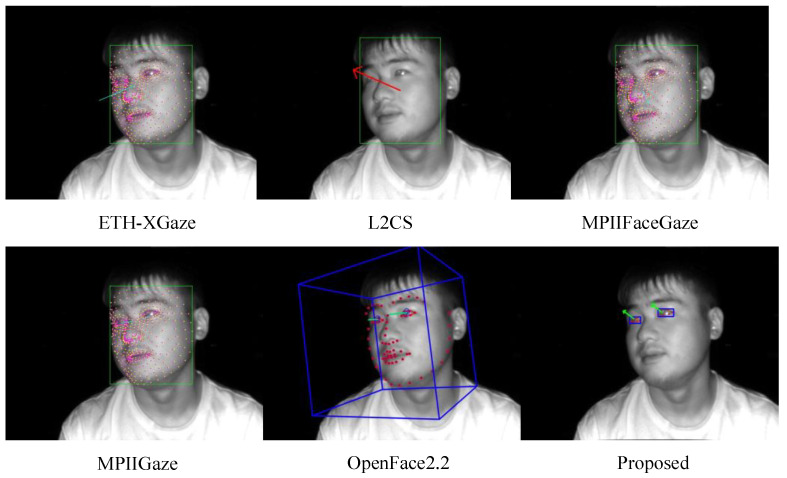
Detection effect of existing state-of-the-art gaze-estimation methods on the IRGD dataset proposed in this study, with arrows and lines indicating the predicted gaze direction of the subject by each model.

In this study, we evaluate the performance of five state-of-the-art gaze-estimation models, namely ETH-XGaze, L2CS, MPIIFaceGaze (also referred to as Spatial Weights CNN), MPIIGaze (also known as GazeNet), and OpenFace 2.2, on our proposed IRGD benchmark dataset for subject gaze detection. These state-of-the-art models were assessed on a local PC, and their effectiveness in detecting the gaze direction of subjects is illustrated in Figure 21. Compared to these state-of-the-art models, the gaze-estimation method proposed in this paper demonstrates the highest accuracy in subject gaze detection. The superior performance is attributed to the proposed method’s robust detection capabilities on low-resolution gaze images and images where the subject is situated at a greater distance from the camera. In gaze images characterized by low resolution, large head pose angles, and a substantial distance between the subject and the camera (≥500 mm), existing state-of-the-art gaze-estimation methods often yield significant discrepancies between the estimated and ground-truth gaze angles due to the blurriness of the image and the limited availability of extractable information from the subject’s eye region. In some instances, the estimated gaze direction may even be the opposite of the subject’s ground-truth line of sight. The gaze-estimation method proposed in this paper overcomes these limitations by utilizing only two eye corner points of a single eye to model the eyeball and employing easily observable pupil points to calculate the gaze angle. Remarkably, even on low-resolution images of 300,000 pixels, our method accurately computes the subject’s gaze angle, maintaining its precision at a distance of 600 mm between the subject and the camera. Our proposed method computes the subject’s gaze angle within the 3D coordinate system of a TOF camera, which significantly mitigates the influence of the head pose on gaze angle estimation. In contrast, existing state-of-the-art gaze-estimation approaches predict the subject’s gaze angle based on 2D images and are highly susceptible to perturbations caused by the subject’s head pose.

In this study, subjects were divided into male and female groups to conduct cross-gender research, aiming to investigate the influence of gender factors on gaze estimation. We found that the aspect ratio of female subjects’ eyes was slightly larger than that of males, but the average gaze angle deviation calculated was close to that of males. The driver gaze detection experiment was conducted within the normal driving distance of 450 mm–600 mm from the dashboard, and the final results confirmed that our proposed gaze-estimation method has stable detection capabilities at different distances. In all experimental results, the maximum gaze angle deviation of the subjects did not exceed 9°, which means that within a driving distance of 600 mm, the maximum detection deviation of our gaze-estimation model in the horizontal and vertical directions is 95 mm. In our Toyota SUV, the distances between various gaze target points inside the car are much greater than this deviation value. Therefore, the detection deviation of the gaze-estimation model proposed in this study is sufficient to distinguish and recognize the driver’s gaze fixation points within the car.

## 6. Conclusions

This paper presents a model-based 3D gaze estimation method using a TOF camera. Unlike most gaze-estimation methods that use RGB images, our gaze-estimation work is completed on low-resolution infrared images, which is extremely advantageous for applications with varying lighting conditions and complex environments, particularly suitable for driver gaze detection in night-time scenarios.

Our main contributions include proposing an infrared gaze dataset (IRGD) for a wide range of free head and eyeball movements and training an eye landmark detection model for the subject’s eye area on the IRGD dataset using YOLOv8. In addition, this study proposes a method to fit a 3D eyeball model using only two corner points of the subject’s single-eye image, enabling our gaze-estimation method to work stably in images where the subject is far from the camera or in low resolution. To address the drawback of the TOF camera not being able to capture the depth information of the subject’s pupil at certain special gaze angles, we solve for the 3D coordinates of the pupil point using the constraint relationship between the subject’s pupil and the center of the eyeball. This paper analyzes the subject’s gaze angle in two directions, horizontal and vertical, and the subject only needs to be calibrated once to calculate the centers of the eyeball and pupil under the horizontal and vertical gaze directions.

Through experimental comparison with other state-of-the-art gaze-estimation models, we have confirmed the effectiveness and advancement of our proposed gaze-estimation method. By comparing with the ground truth gaze angle, the maximum detection deviation of the gaze-estimation method proposed in this paper is less than 9° for the subjects. Using RMSE as the performance evaluation standard, our gaze-estimation method has detection deviations of 6.03° and 4.83° in the horizontal and vertical directions, respectively. We employed the proposed gaze-estimation method to detect gaze angles in subject images with a resolution of 640 × 480. The model proposed in this paper achieved a detection speed of 7.8 frames per second (FPS) when implemented on a local PC equipped with an Intel Core i7-11800H @ 2.30GHz CPU. We also evaluated the accuracy of our gaze-estimation method for driver gaze detection in real car driving scenarios. In future research, we plan to improve the robustness of the gaze-estimation model to extreme head poses and self-occlusion. We will explore the possibility of dynamically selecting the subject’s left or right eye in conjunction with the subject’s head pose to enhance the accuracy of gaze estimation.

## Figures and Tables

**Table 1:** The angle calculation results of gaze estimation based on the 3D eyeball center localization method mentioned in [30].

Number	Ground-Truth Yaw Angle [Degree]	Distance from Pupil to TOF Camera [mm]	Calculated Yaw Angle Value [Degree]	Absolute Deviation [Degree]
1	41.6	495.29	30.7	**10.9**
2	27.01	502.35	33.19	6.18
3	8.39	495.29	9.83	1.44
4	−12.3	498.82	−12.93	0.63
5	−30.39	498.82	−24.13	6.26
6	−43.54	502.35	−52.93	9.39

**Table 2:** The radius value of the 3D eyeball model fitted using the method from [36].

The Distance from the Subject’s Head to the TOF Camera [mm]	The Depth Values of Each Marked Point on the Depth Image [mm]								The Radius Value of the Eyeball [mm]
	1	2	3	4	5	6	7	8	
≈200	184.3	184.3	184.6	188.2	183.7	188.2	184.3	185.6	11.8
≈300	274.5	274.5	273.2	274.4	278.4	279.1	278.4	274.5	12.5
≈400	360.8	360.8	361.2	362.2	356.9	361.2	356.9	360.8	11.5

**Table 3:** Comparison and summary of current open-source datasets and our proposed IRGD dataset.

Datasets	With Face	Total	Gaze Pitch	Gaze Yaw	Image Types
MPIIGaze	No	213 K images	±20°	±20°	RGB
MPIIFaceGaze	Yes	213 K images	±20°	±20°	RGB
EYEDIAP	Yes	94 Videos	±30°	±40°	RGB and RGBD
UnityEyes	No	User defined	User defined	User defined	RGB
ETH-XGaze	Yes	Over 1 M images	±70°	±120°	RGB
ManiGaze	Yes	11 K images (from videos)	±40°	±50°	RGB and RGBD
Gaze360	Yes	172 K images	−50°	±140°	RGB
Columbia	Yes	5 K	±10°	±15°	RGB
EVE	Yes	Over 12 M images (from videos)	±50°	±60°	RGB
GazeCapture	Yes	Over 2 M images	±20°	±20°	RGB
UT Multi-view	No	64 K	±36°	±50°	RGB
SynthesEyes	No	11 K	±25°	±35°	RGB
IRGD	Yes	150 K	±50°	±50°	IR and Depth

## Data Availability

The data presented in this study are available on request from the corresponding author. The data are not publicly available due to privacy protection for data recorders.
